# Effects of Different Dietary and Lifestyle Modification Therapies on Metabolic Syndrome in Prediabetic Arab Patients: A 12-Month Longitudinal Study

**DOI:** 10.3390/nu10030383

**Published:** 2018-03-20

**Authors:** Hanan A. Alfawaz, Kaiser Wani, Abdullah M. Alnaami, Yousef Al-Saleh, Naji J. Aljohani, Omar S. Al-Attas, Majed S. Alokail, Sudhesh Kumar, Nasser M. Al-Daghri

**Affiliations:** 1 Prince Mutaib Chair for Biomarkers of Osteoporosis, Biochemistry Department, College of Science, King Saud University, Riyadh 11451, Saudi Arabia; halfawaz@ksu.edu.sa (H.A.A.); wani.kaiser@gmail.com (K.W.); aalnaami@yahoo.com (A.M.A.); omrattas@ksu.edu.sa (O.S.A.-A.); msa85@yahoo.co.uk (M.S.A.); 2 Department of Food Science and Nutrition, College of Food Science & Agriculture, King Saud University, Riyadh 11451, Saudi Arabia; 3 Biomarkers Research Program, Biochemistry Department, College of Science, King Saud University, Riyadh 11451, Saudi Arabia; 4 College of Medicine, King Saud bin Abdulaziz University for Health Sciences, Riyadh 11461, Saudi Arabia; alaslawi@hotmail.com; 5 Specialized Diabetes and Endocrine Center, King Fahad Medical City, Faculty of Medicine, King Saud bin Abdulaziz University for Health Sciences, Riyadh 11525, Saudi Arabia; najijohani@gmail.com; 6 Division of Metabolic and Vascular Health, Clinical Sciences Research Institute, University Hospitals Coventry and Warwickshire Trust, Walsgrave, Coventry CV2 2DX, UK; Sudhesh.Kumar@warwick.ac.uk

**Keywords:** impaired glucose regulation, lifestyle modifications, metabolic syndrome, type 2 diabetes, metformin

## Abstract

This three-arm, randomized, controlled study aimed to determine the differences in the effects of general advice (GA) on lifestyle change, intensive lifestyle modification programme (ILMP) and GA + metformin (GA + Met) in reducing the prevalence of full metabolic syndrome (MetS) in subjects with prediabetes; 294 Saudis with prediabetes (fasting glucose 5.6–6.9 mmol/L) were initially randomized, 263 completed 6 months and 237 completed 12 months. They were allocated into three groups: GA group which received a standard lifestyle change education; ILMP which followed a rigorous lifestyle modification support on diet and physical activity; and a GA + Met group. Anthropometric and biochemical estimations were measured. Full MetS (primary endpoint) and its components (secondary endpoint) were screened at baseline, 6 and 12 months. Full MetS in the ILMP group decreased by 26% (*p* < 0.001); in GA + Met group by 22.4% (*p* = 0.01) and in GA group by 8.2% (*p* = 0.28). The number of MetS components decreased significantly in the ILMP and GA + Met groups (mean change 0.81, *p* < 0.001 and 0.35, *p* = 0.05, respectively). Between-group comparison revealed a clinically significant decrease in MetS components in favor of the ILMP group (−0.58 (−0.88–0.28), *p* < 0.001). This study highlights the clinical potency of ILMP versus other diabetes prevention options in reducing MetS in Saudi adults with elevated fasting glucose.

## 1. Introduction

Type 2 diabetes mellitus (T2DM) has a huge impact on the health status of patients and the overall health care cost of the country. One big opportunity to reduce such an impact is to reduce the incidence of the disease by focusing on high-risk people such as those with impaired glucose regulation. This condition is characterized either by impaired fasting glucose (IFG) (fasting glucose levels 5.6–6.9 mmol/L) or impaired glucose tolerance (IGT) (2-h oral glucose tolerance test (OGTT) 7.8–11 mmol/L) [1]. Every year, about 5–10% of people with IGT progress to T2DM [2]. Fortunately, landmark clinical trials like the Diabetes Prevention Programme (DPP) [3] and others [4,5] have revealed the effectiveness of lifestyle modifications in reducing the incidence of T2DM. The major focus of these interventions were weight loss and physical activity. However, a recent meta-analysis [6,7] concluded that when translated into routine clinical settings, these expensive programmes have more effect on weight reduction and less effect on diabetes risk reduction. Clearly, such intervention studies need to be done, not only to add to the literature to better predict the outcomes on a global health-care perspective but also to devise large-scale effective and low cost intervention programmes on a regional level such as in Saudi Arabia, where data are limited.

Identification of individual risk factors associated with the increased effectiveness of lifestyle intervention programmes may help in devising them in a more economical and practical way. Metabolic Syndrome (MetS), an amalgam of cardiovascular risk factors, is a major health problem globally. It is defined by the National Cholesterol Education Program Adult Treatment Panel III (NCEP ATP III) as the presence of at least three out of five risk factors (central obesity, hyperglycemia, low HDL-cholesterol, hypertriglyceridemia and elevated blood pressure) [8]. The prevalence of MetS is increasing rapidly worldwide, including Saudi Arabia, as a result of rapid economic growth and Westernization of diet [9,10].

The risk of developing incident T2DM in patients with MetS is manifold compared to those without this condition [11]. Also, all components of MetS are independent risk factors for developing T2DM [12]. Prevention of T2DM in subjects with impaired glucose regulation through lifestyle modifications in diet, physical activity or by giving drugs like metformin have been studied in the past, however, the applicability of these treatments in prevention or reversal of MetS is largely unknown, particularly in a population like Saudi Arabia where the prevalence of both T2DM and MetS is high [13,14]. We hypothesize that lifestyle modifications in diet and physical activity have a role in preventing or reversal of Mets in subjects with impaired glucose regulation. Hence, this study aimed to investigate the differences in the effectiveness of lifestyle modifications (diet and physical activity) and drug therapy (metformin), in an adult Saudi population with impaired glucose regulation, in preventing or reversing full MetS and its individual components.

## 2. Materials and Methods

This is a 12 months, 2-center, 3-arm randomized controlled (1:1:1), lifestyle intervention study conducted from April 2013 until March of 2017. This lifestyle intervention programme was approved by the Ethics Committee of the College of Science, King Saud University; Riyadh, Saudi Arabia (Reference# 8/25/220355) and funded by the National Plan for Science and Technology; Riyadh, Saudi Arabia (Grant# 12-MED2881-02). All procedures followed were in accordance with the ethical standards of the responsible committee on human experimentation (institutional and national) and with the Helsinki Declaration of 1975, as revised in 2008. Written informed consent was obtained from each participant prior to inclusion in this 12-month interventional study.

### 2.1. Study Population

A total of 294 Saudi males and females (age range 25–60) attending King Khalid University Hospital and King Salman Hospital in Riyadh, Saudi Arabia, agreed to take part in this lifestyle intervention Programme. The criteria for selection was a fasting glucose level of 5.6 to 6.9 mmol/L, identified as one of the five components of MetS (NCEP ATP III criteria) [15]. Interested participants were referred to the concerned physician who used the guidelines stated under section “prevention or delay of type 2 diabetes” in “standards for medical care in diabetes” [16] to screen the candidates. Subjects who were already on anti-hyperglycemic treatment; pregnant or lactating women; with known renal, hepatic, pulmonary, cardiac, etc., complications were excluded.

**Figure 1:**
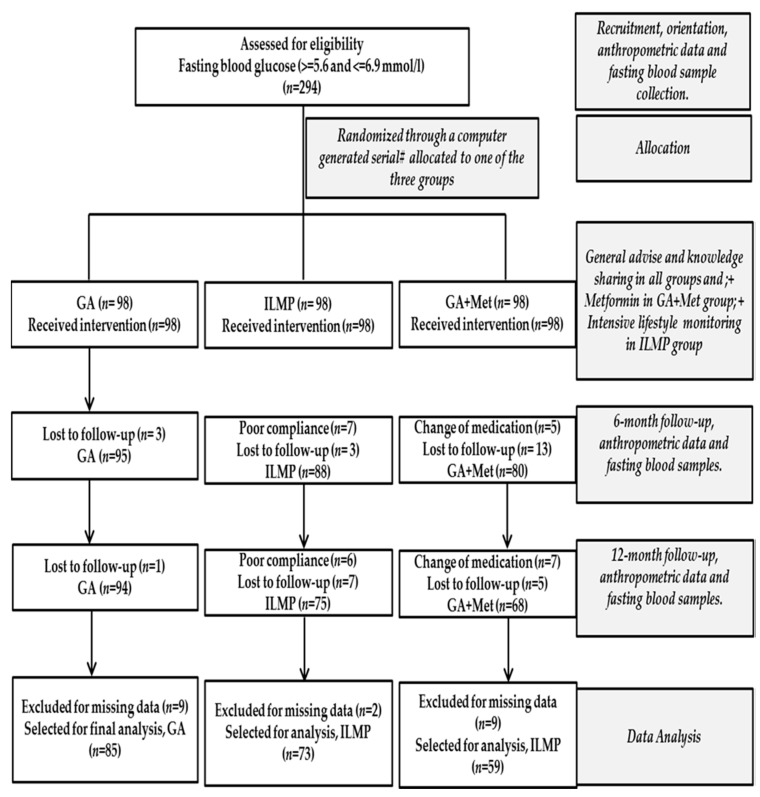
Flow chart detailing the participation of subjects and their allocation to treatment groups.

A computer-generated serial number, randomly assigned to one of the three intervention groups (GA, ILMP, and GA + Met), was given blindly to each participant. All participants were allocated (1:1:1) to receive one of the three interventions. True allocation concealment was done since the research personnel involved cannot adjust randomization. The total duration of this lifestyle intervention programme was 12 months. Fasting blood samples and anthropometric data was collected at recruitment, at 6-month and at the end of the programme. Out of the 294 initially recruited, the data for 217 was used in this study. A flow chart summarizing the programme is provided in Figure 1.

### 2.2. Intervention

All participants had an orientation session with concerned physician and a dietician where they were educated about risk of developing T2DM and the current scenario of diabetes worldwide and Saudi Arabia. They were advised to adopt lifestyle modifications in their dietary habits; weight reduction; exercise; increased physical activity etc. This knowledge sharing included distributing pamphlets and booklets with information related to lifestyle modifications in earlier programmes, done elsewhere [17,18]. In addition to this, every four months, seminars were conducted at the auditorium of the respective hospitals in which the investigators of the current study educated the participants about lifestyle modifications to prevent T2DM. This intervention process was unified across the two study centers.

The participants in the general advice (GA) group received the usual instructions to lifestyle change as described above. In addition to this the participants in intensive lifestyle modification programme (ILMP) group were followed with a rigorous lifestyle modification support published earlier [19,20], which included:

(a) Individual consultation with the dietician was done to assess the participant’s food intake. Special dietary charts were supplied to participants explaining how to reduce the total fat intake to less than 30% of energy consume and increase the fiber intake to 15 g/1000 Kcal.
(b) Guidelines for physical activity were supplied as pamphlets to each participant. Also, each participant was given a pedometer (081564483, Patterson Medical) and recommended at least 5000 steps per day to gradually increase as tolerance develops.
(c) Individual consultation with an expert on vitamin D to educate about the benefits of optimal levels of vitamin D for good health. They were recommended to expose to sunlight for at least 30 min either before 10 a.m. and/or after 3 p.m. twice a week.

The intervention in ILMP group was monitored regularly which were scheduled every three months during the course of the programme.

The third intervention group (GA + Met) was provided with the same general advice and were given 500 mg of metformin hydrochloride, twice a day. The participants in ILMP and GA + Met groups were regularly contacted by research assistants to reinforce instructions through phone calls. 

### 2.3. Anthropometric and Biochemical Measurements

Anthropometrics were collected at recruitment (baseline), 6-month and 12-month. The anthropometrics included height (cm), weight (kg), waist and hip circumferences (cm), systolic and diastolic blood pressure by standard methods. Fasting blood samples, taken at each time point, were sent immediately to Prince Mutaib Chair for biomarkers in Osteoporosis (PMCO), King Saud University (KSU), Riyadh where they were processed, aliquoted and stored at recommended temperature for further analysis.

Fasting blood glucose and lipid profile was quantified using routine biochemical tests in an automated biochemistry analyzer (Konelab 20, Thermo-Fischer scientific, Helsinki, Finland). The reagents were supplied ready to use by Thermo Fischer (catalog# 981379 for glucose; 981812 for total cholesterol; 981823 for HDL-cholesterol and 981301 for triglyceride). The imprecision, calculated as the total CV, was ≤5%, ≤3.5%, ≤4% and ≤4% for these tests respectively. 25(OH)vitamin D was quantified using COBAS e-411 autoanalyzer (Roche Diagnostics, Indianapolis, IN, USA). Glycated haemoglobin (HbA1c) was quantified in DCA vantage analyzer (Siemens, Munich, Germany). The imprecision of the HbA1c assay was ≤3.6% in the important clinical ranges. The standards and controls used for these biochemical assays were routinely tested by Quality assurance department of KSU, for highly reproducible research data.

### 2.4. Outcome Variables

For the purpose of this study, the status of MetS and its five components were evaluated at follow up (6-month and 12-month) versus baseline. MetS was defined by the criteria set in “The National Cholesterol Education Programme Adult Treatment Panel III” (NCEP ATP III) as having atleast three of the five components [15]:

(a) Central obesity-waist circumference of >101.6 cm in males and >88.9 cm in females.
(b) Hyperglycemia-fasting glucose > 5.6 mmol/L.
(c) Low HDL-Cholesterol < 1.03 mmol/L in males and <1.30 mmol/L in females.
(d) Hypertriglyceridemia-fasting triglycerides > 1.7 mmol/L.
(e) Hypertension-systolic blood pressure > 130 mmHg and/or diastolic blood pressure > 85 mmHg.

Two other variables were tested in this study for the intervention effects between groups. One was the total number of MetS components (taken as a continuous variable) and the other was the MetS risk-score. The MetS risk-score was constructed for the evaluation of continuous MetS status, calculated using the formula with cut-off values employed to define each component of MetS, with consideration to age and gender as follows:

MetS risk-score = ((waist/101.6 for males or 88.9 for males) + (fasting glucose/5.6) − (HDL-Cholesterol/1.03 for males or 1.30 for females) + (triglyceride/1.7) + (systolic BP/130) + (diastolic BP/85)) × (Age/45 for males or 50 for females).

The Receiver Operating Characteristic (ROC) analysis was employed to test this score for predicting MetS in our data, with full MetS positive (≥3 MetS components) versus full MetS negative (<3 MetS components). The ROC revealed an area under the curve (AOC) of 0.890 with 95% confidence interval of 0.86 and 0.92 and a *p*-value of <0.001. The cut-off of MetS risk-score for predicting MetS, obtained in ROC analysis, was 3.85 (Supplementary Figure S1, Table S1–S2).

### 2.5. Data Analysis

As expected with longitudinal studies, the data in this study had random missing values which are a limitation for utilizing any test on repeated measure data. Hence, the missing data (<5% of the total data points in any variable) was dealt with the last observation carried forward (LOCF) method. However, as much as possible, the LOCF was minimized by removing the data of the subjects lost to follow up at 6-month or 12-month and also by removing ones with >5% missing data in any variable (Figure 1). The remaining data (*n* = 217) was analyzed using SPSS 21. Continuous normally distributed variables were summarized as mean ± standard deviation while median (25th percentile, 75th percentile) was used for continuous non-normal variables. Simple One-way ANOVA and Kruskal-Wallis one-way ANOVA were used to test the differences between the three treatment groups at baseline. The status of MetS and its five components were evaluated as present/absent at all three time-points, which were presented as frequency (% of the present in the respective group) and chi-square test (McNemar 2 × 2 contingency table) was used to calculate the *p*-value of the difference in percentages. The intervention effect within each group was shown as Odds ratio (95% confidence interval) and respective *p*-value representing odds of having MetS and its components independently at follow-up compared to baseline and this data was generated by Generalized Estimating Equation (GEE) in SPSS for repeated measures of nominal data. Finally, the intervention effect between the groups was shown as mean Change (95% confidence interval), *p*-value for the total number of MetS components (taking MetS components as scalar quantity) and MetS risk-score by mixed repeated measures ANCOVA. *p*-Values were considered significant at <0.05.

## 3. Results

A total of 294 (98 in each group) Saudi adults with impaired fasting glucose were initially randomized and 237 (94 in GA, 75 in ILMP and 68 in GA + Met) completed the entire 12 months of this intervention. The most common reasons for drop out included loss to follow-up and poor compliance. After excluding persons with missing data >5% in any parameter, the data for 217 subjects (85 in GA, 73 in ILMP and 59 in GA + Met) were used for analysis (Figure 1).

### 3.1. Anthropometric and Biochemical Characteristics at Baseline and over Time

Table 1 shows the anthropometric, glycemic, and lipid characteristics of the study participants at baseline, 6-month, and 12-month, according to treatment groups. The mean change in fasting glucose from baseline to end of study for ILMP and GA + Met groups decreased significantly (−0.39 mmol/L, *p* = 0.003 and −0.81 mmol/L, *p* < 0.001 respectively). This was not observed in the GA group (−0.005 mmol/L, *p* = 0.65). Weight was significantly reduced in the GA + Met group from baseline to 12 months (mean change of −4.15 kg, *p* < 0.001). ILMP group also showed a significant reduction in weight (mean change = −1.86 kg, *p* = 0.015) while the average weight for GA group increased from baseline to the end of the study by 0.49 kg. Waist, systolic blood pressure and triglycerides significantly reduced from baseline to 12 months in the ILMP group (−1.61 cm, *p* = 0.004; −2.59 mmHg, *p* = 0.049 and −0.23 mmol/L, *p* = 0.03 respectively). At baseline, the three treatment groups were significantly different in waist (*p* < 0.01), systolic blood pressure (*p* = 0.01), diastolic blood pressure (*p* = 0.02), fasting glucose (*p* < 0.01), HbA1c (*p* < 0.01) and vitamin D levels (*p* = 0.03).

### 3.2. Prevalence of MetS and Its Components at Baseline and Overtime

Table 2 shows the percentage of subjects having different components of MetS and full MetS in the three treatment groups at baseline, follow-up and percentage of subjects in which there was a change in status over time. MetS and its components were evaluated as binomial variables. Component 1 (central obesity) and component 4 (hypertriglyceridemia) showed the lowest changes at follow-up in all the three groups (−1.2%, −1.4% and −1.7% in central obesity; and +3.5%, −4.1% and 0% in hypertriglyceridemia for groups GA, ILMP and GA + Met respectively). Component 3 (Low HDL-Cholesterol) increased by 7.1% (*n* = 6) in GA group at end of the study compared to baseline while it decreased by 6.8% (*n* = 5) and 3.4% (*n* = 2), respectively, in ILMP and GA + Met groups. Component 5 (Hypertension) increased by 2.4% (*n* = 2) and by 8.5% (*n* = 5, *p* = 0.035) in GA and GA + Met groups, respectively, while it decreased by 1.4% (*n* = 1) in the ILMP group. The highest change was seen in component 2 (hyperglycemia) where from baseline to end of the study, hyperglycemia was reduced by 22.4% (*n* = 19), 38.4% (*n* = 28) and 39% (*n* = 23), respectively, in GA, ILMP, and GA+ILMP groups respectively. Full MetS was also significantly reduced by 8.2% (*n* = 7), 26% (*n* = 19, *p* < 0.001), and 22.4% (*n* = 13, *p* = 0.013), respectively, in GA, ILMP, and GA + Met groups.

### 3.3. Odds of Having MetS and Its Components at Follow-Up Compared to Baseline

Table 3 shows the odds of having MetS and its individual components at follow-up compared to baseline in each group. Supplementary Table S3 shows the odds of having MetS for these covariates, independent of each other.

The odds of central obesity and hypertriglyceridemia showed marginal changes in all groups, with odds ratio (95% confidence interval (C.I.)) of 0.84 (0.4, 1.8) and 0.86 (0.5, 1.3), respectively, in GA group; 0.80 (0.3, 2.2) and 0.80 (0.5, 1.4) in ILMP group and 0.74 (0.2, 3.5) and 1.0 (0.6, 1.8) in GA + Met group. The odds of low HDL-cholesterol increased (1.66 (0.8, 3.5)) in GA group, but was reduced both in ILMP (0.79 (0.4, 1.4)) and GA + Met (0.86 (0.4, 1.7)) groups. The odds of hypertension increased in GA + Met group (2.36 (1.2, 4.6), *p*-value = 0.012) and GA group (1.28 (0.5, 3.1)) but was modestly decreased in ILMP group (0.88 (0.4, 2.1)). The odds of full MetS was significantly reduced both in ILMP (0.25 (0.1, 0.4), *p*-value < 0.001) and GA + Met (0.32 (0.1, 0.7), *p*-value = 0.005) groups only.

**Figure 2:**
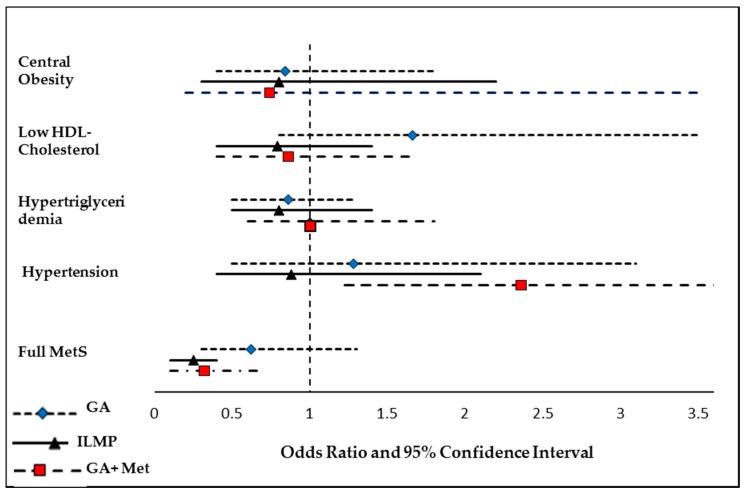
Odds ratio (OR) of MetS and its components for three treatment groups. The model is adjusted for age, sex, BMI and baseline covariates: waist, systolic and diastolic BP, fasting glucose, HbA1c, and vitamin D.

Figure 2 shows the odds ratio of MetS and its components at the end of the study compared to baseline for different treatment groups. The odds for MetS were significantly reduced in ILMP group followed by GA + Met group.

### 3.4. Intervention Effects in Total Number of MetS Components and MetS Risk Factor

Table 4 shows the intervention effects in the total number of MetS components and the MetS risk-score. The number of MetS components significantly decreased from baseline to the end of the study in the ILMP group (mean change (standard error)) of 0.81 (0.13), *p*-value < 0.001. Similarly, it decreased in GA + Met group by 0.35 (0.18), *p*-value = 0.05. Between-group comparison revealed that the decrease in the number of MetS components in ILMP was statistically more significant than GA (*p* < 0.001). Between (GA + Met) and GA groups, this decrease was not significant. Intervention effects between groups for MetS risk score showed the same trend between ILMP vs. GA group (0.31 (−0.53, −0.09); *p* = 0.003).

**Figure 3:**
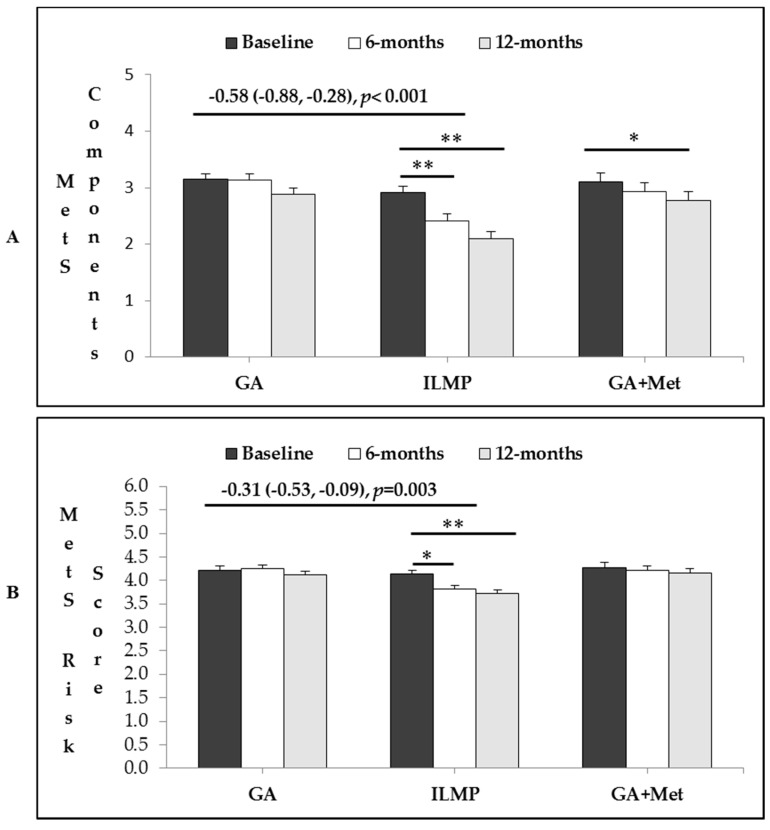
Intervention effects in total number of MetS components (**A**) and MetS risk-score (**B**) for three treatment strategies calculated at baseline and follow-up. Data is adjusted for age, sex and BMI, systolic and diastolic BP, waist, fasting glucose, HbA1c, and vitamin D (all baseline values). The overall change in ILMP and GA + Met groups versus GA group reported as Mean Change (95% confidence interval). Within group intervention effect is shown as * for *p* < 0.05 or ** for *p* < 0.01.

Figure 3 shows the intervention effects of the three treatment strategies on MetS, represented by bar graphs showing changes in total number of MetS components and MetS risk-score overtime.

## 4. Discussion

In this study, Saudi adults with impaired glucose regulation were given treatment based on lifestyle modifications in diet and exercise or low doses of metformin to assess the status of MetS and its individual components over time. Changing lifestyle and reducing the burden of chronic diseases in this population is of great public health importance, however, the impact of such programmes on full MetS and components of MetS in the pre-diabetic Saudi population has not been studied. It is notable that, for the past two decades since these lifestyle intervention studies started, the study outcome variables in most of them has been the incidence of diabetes. In this study, we chose a broader set of outcome measures and focused on the status of MetS and its components together, as well as those independently considered as risk factors for the incidence of T2DM. To the best of our knowledge, this is the first such study in Saudi Arabia which emphasizes the changes in the status of MetS and its components through lifestyle modifications. A similar study was done by the Diabetes Prevention Program (DPP) research group in 2005 [21] and focused on lifestyle intervention and metformin-based therapies for MetS.

Mean fasting glucose was significantly reduced in the ILMP and GA + Met groups while in the GA group this reduction was modest. Similarly, the prevalence of hyperglycemia was reduced significantly by 38.4% and 39% in the ILMP and GA + Met groups, respectively. This improvement in mean fasting glucose is similar to previous studies done in USA [22], Italy [23], and Iran [24] where, on an average, fasting glucose was reduced by 6 mg/dl from baseline to the end of the study in the dietary intervention group. Lifestyle modifications, including replacing high-glycemic index diet with low-glycemic index diet like vegetables, dairies, and whole grains, etc. might have played a role in the reduction in mean fasting glucose [25]. However, as expected, the mean reduction in fasting glucose in the ILMP group is lesser than that found in the GA + Met group. Though the molecular mechanism of the action of metformin remains debated, a consensus is on the direct action of metformin on hepatic glucose production and improving insulin sensitivity in muscle and fat cells [26]. Furthermore, metformin’s action is not limited to glucose reduction and insulin sensitivity, it is also an effective weight loss drug due to its effect on appetite and its regulation of fat oxidation, storage in liver and adipose tissue [27]. In our study, the mean weight loss from baseline to the end of the study was 4.15 kg in GA + Met group and this was higher than the ILMP group (1.86 kg).

The ILMP group showed modest improvements in all of the other components of MetS, namely, central obesity, low HDL-cholesterol, hypertriglyceridemia and hypertension. Waist circumference also decreased from baseline to the end of study in the ILMP group by an average of 1.86 cm. This change in waist circumference is close to a mean decrease of 2.7 cm found in a meta-analysis of six interventions [28]. The odds for low HDL-cholesterol was 1.66 (95% confidence interval 0.8 to 3.5) in the GA group, indicating a higher risk in the absence of an intensive lifestyle change program. Similarly, the odds for hypertriglyceridemia at the end of the study versus baseline in the metformin group was 1.0 (0.6, 1.8), which together with the odds obtained for low HDL-cholesterol indicated that GA + Met induce less effects on lipid levels than the ILMP. This was in agreement with studies like [29] which reported significantly larger improvement in lipid indexes in the ILMP group than in the GA + Met group.

The prevalence of hypertension was significantly higher at the end of the study compared to baseline in the GA + Met group (O.R. 2.36 (1.2, 4.6), *p*-value 0.012). The literature on the effect of metformin on hypertension has yielded conflicting results. While some [30] suggest metformin has no intrinsic effect on blood pressure, others, like a recent meta-analysis from 26 studies [31], suggested that metformin could effectively lower systolic blood pressure especially in those with IGT. There are also reports on the role of metformin in reversing pulmonary hypertension through inhibition of aromatase synthesis [32]. In any case, the findings in this study related to hypertension in the GA + Met group should be looked at keeping in mind that most of the study participants (72.3% of all subjects and 71.2% of GA + Met group) are females. Women normally tend to have lower blood pressure than men, related to sex differences in concentrations of angiotensin II and sodium reabsorption in distal nephron [33].

The decrease in the prevalence of full MetS at the end of the study compared to baseline was greater in ILMP (−26%, *p*< 0.001) and GA + Met groups (−22.4%, *p* = 0.013) than the GA group (−8.2%, *p* = 0.281). Furthermore, the analysis of the number of MetS components as well as MetS risk-score suggests that although ILMP was superior to GA + Met, both are more effective compared to GA. The biological mechanism whereby ILMP or metformin exerts its protective effect from MetS is complex and unclear; however, greater intakes of dietary fibers, low-glycemic index foods, vitamins, more physical activity, etc. in ILMP all contribute to the reduction in low-grade inflammation, normally associated with MetS [34,35].

The MetS risk-score, employed in testing the hypothesis of the study regarding MetS reversal/reduction with three different treatments, had high discriminative power for presence/absence of MetS (AOC of 0.890, 95% C.I. of 0.86 to 0.92 and a *p*-value of <0.001, Supplementary Figure S1, Tables S1–S2). A continuous risk-score for predicting MetS has been developed by approaches such as taking standardized residuals (z-scores) or scores from principal component analysis of risk factors [36,37]. These scores, although precise, are complex and require specialized statistical software. While the authors attempted an easy-to-calculate continuous MetS risk-score, it should be used with caution as the values apply to the population and local laboratory reference values.

The authors acknowledge certain limitations of the study. Firstly, the study was done on subjects with IGT and, hence, the results may not hold true for those with normal glucose regulation but having full MetS manifestations. Such intensive lifestyle change programmes should be conducted in these categories to have a better understanding of the benefits. Secondly, the study does not give details about the actual changes in lifestyle that each participant in this programme carried out in terms of variables related to diet or physical activity, measured before intervention and follow up. Despite the study being based on self-monitoring; it showed a significant reversal in MetS in the ILMP group which suggests that, if guided properly, people are willing to change from a sedentary to a healthy lifestyle. The study also offered a unique statistical approach in assessing MetS and its components and by evaluating the change in the total number of MetS components as an outcome variable. Also, the MetS risk-score devised in this study, if further researched, may provide a simplistic estimate for assessing MetS in an individual.

## 5. Conclusions

Intensive lifestyle modifications or low dose metformin for a period of 12 months significantly reduces MetS manifestation in individuals with prediabetes, with lifestyle modifications being superior to metformin, as the latter’s potency is limited to weight loss and reduction of hyperglycemia, while the former improves all the components of MetS together as well as independently.

## Figures and Tables

**Table 1:** Anthropometric and biochemical characteristics at baseline and follow-up.

Treatment (*n*) Female/Male	GA (85) 64/21	ILMP (73) 51/22	GA+Met (59) 42/17	*p* ^B^
**Anthropometrics**				
Age (years)	42.3 ± 11.2	43.4 ± 7.8	42.6 ± 6.9	0.74
**Weight (Kg)**				
Baseline	81.7 ± 13.9	79.6 ± 15.9	80.4 ± 14.9	0.67
6-month	82.3 ± 13.9	78.7 ± 15.9	77.6 ±13.9	
12-month	82.2 ±13.4	77.7 ±16.2	76.3 ±14.1	
Change at 6 months	0.61	−0.93	−2.86 **	
Change at 12 months	0.49	−1.86 *	−4.15 **	
**BMI (kg/m^2^)**				
Baseline	32.6 ± 5.8	31.3 ± 6.4	32.1 ± 5.7	0.18
6-month	32.8 ± 5.9	31.0 ± 6.7	31.0 ± 5.4	
12-month	32.8 ± 5.7	30.6 ± 6.6	30.4 ± 5.3	
Change at 6 months	0.26	−0.32	−1.14 **	
Change at 12 months	0.21	−0.71 *	−1.68 **	
**Waist (cm)**				
Baseline	95.6 ± 6.8	97.9 ± 13	103.6 ± 12.5	**<0.01**
6-month	95.7 ± 6.7	97.7 ± 13.5	102.6 ± 12.8	
12-month	95.5 ± 6.2	96.3 ± 13	102.6 ± 12.3	
Change at 6 months	0.12	−0.25	−0.95	
Change at 12 months	−0.09	−1.61 **	−0.96	
**Hips (cm)**				
Baseline	110.2 ± 7.9	111. 9 ± 12	111.0 ± 12	0.60
6-month	110.5 ± 7.9	111.0 ± 11.6	109.5 ± 10.9	
12-month	110.6 ± 7.6	109.9 ± 12.2	109.6 ± 10.9	
Change at 6 months	0.26	−0.86 *	−1.48 **	
Change at 12 months	0.44	−1.98 **	−1.39 *	
**Systolic BP (mmHG)**				
Baseline	120.0 ± 12.1	122.1 ± 15.8	127.4 ± 11.6	**0.01**
6-month	117.8 ± 14.4	120.0 ± 18.5	129.2 ± 11.1	
12-month	119.2 ± 15.8	119.5 ± 16.6	129.3 ± 10.7	
Change at 6 months	−2.25	−2.12	1.86	
Change at 12 months	−0.86	−2.59 *	1.97	
**Diastolic BP (mmHG)**				
Baseline	76.4 ± 9.7	76.0 ± 11.9	80.6 ± 9.0	**0.02**
6-month	76.4 ± 12.1	76.0 ± 12.1	81.2 ± 11.1	
12-month	77.1 ± 13.7	74.6 ± 12.8	83.3 ± 9.2	
Change at 6 months	0.06	0.14	0.68	
Change at 12 months	0.71	−1.38	2.73	
**Glycemic Profile**				
**Fasting Glucose (mmol/L)**				
Baseline	6.0 ± 0.4	6.1 ± 0.4	6.6 ± 0.5	**<0.01**
6-month	6.1 ± 0.7	5.7 ± 0.8	6.0 ± 1.3	
12-month	5.9 ± 0.9	5.7 ± 0.8	5.8 ± 1.7	
Change at 6 months	0.09	−0.40 **	−0.56 **	
Change at 12 months	−0.05	−0.39 **	−0.81 **	
**HbA1c**				
Baseline	5.6 ± 0.5	5.8 ± 0.4	5.6 ± 0.5	**<0.01**
6-month	5.7 ± 1.6	5.6 ± 0.4	5.1 ± 1.5	
12-month	5.6 ± 1.5	5.5 ± 1.0	5.0 ± 1.7	
Change at 6 months	0.06	−0.22	−0.47 **	
Change at 12 months	−0.06	−0.30	−0.53 **	
**Lipid Profile**				
**Total Cholesterol (mmol/l)**				
Baseline	4.8 ± 1.0	5.2 ± 1.3	4.8 ± 1.2	0.06
6-month	4.8 ± 1.2	5.0 ± 1.1	4.9 ± 1.0	
12-month	4.6 ± 1.1	5.0 ± 1.0	4.9 ± 1.2	
Change at 6 months	−0.05	−0.19	0.04	
Change at 12 months	−0.22	−0.24	0.06	
**HDL-Cholesterol (mmol/l)**				
Baseline	1.1 ± 0.3	1.2 ± 0.4	1.2 ± 0.4	0.49
6-month	0.93 ± 0.4	1.2 ± 0.4	1.1 ± 0.4	
12-month	0.96 ± 0.4	1.2 ± 0.4	1.2 ± 0.3	
Change at 6 months	−0.16	0.02	−0.02	
Change at 12 months	−0.13	0.03	0.01	
**Triglycerides (mmol/l)**				
Baseline	1.4 (1.1, 2.1)	1.5 (1.1, 1.8)	1.6 (1.2, 2.0)	0.75
6-month	1.5 (1.1, 2.1)	1.3 (1.1, 1.8)	1.6 (1.3, 2.2)	
12-month	1.4 (1.1, 2.0)	1.2 (1.0, 1.7)	1.6 (1.3, 2.0)	
Change at 6 months	0.11	−0.12	0.03	
Change at 12 months	−0.03	−0.23 *	0.00	
**25(OH) vitamin D (nmol/l)**				
Baseline	41.7 (24.0, 73.0)	47.3 (30, 67.2)	57.0 (38, 96)	**0.03**
6-month	45.0 (28.5, 72.6)	54.1 (40, 68)	62.4 (41, 95)	
12-month	48.3 (28.3, 74.7)	56.1 (40, 72.2)	62.4 (42, 96)	
Change at 6 months	0.66	3.78	0.98	
Change at 12 months	2.49	5.82	3.79	

Note: Data presented as Mean ± SD for continuous normal variables and medians (25th–75th percentile) for continuous non-normal variables; * and ** represent significant mean change at *p* < 0.05 and *p* <0.01 respectively. *p* ^B^ represents difference between treatment groups at baseline. GA is “general advice group”, ILMP is “intensive lifestyle monitoring programme group”, GA + Met is “Metformin group”, BMI is “body mass index”, BP is “blood pressure”, HbA1c is “glycated haemoglobin”, HDL is “high density lipoprotein”. *p* < 0.05 is considered significant.

**Table 2:** Prevalence of MetS and its 5 components at baseline and follow-up.

		Central Obesity	Hyperglycemia	Hyper Triglyceridemia	Low HDL-Cholesterol	Hypertension	MetS
GA (85)	Baseline	63 (74.1)	85 (100)	64 (75.3)	36 (42.4)	14 (16.5)	62 (72.9)
	6-month	61 (71.8)	76 (89.4)	71 (83.5)	36 (42.4)	19 (22.4)	63 (74.1)
	12-month	62 (72.9)	66 (77.6)	70 (82.4)	33 (38.8)	16 (18.8)	55 (64.7)
	% change-6	−2.3	−10.6	8.2	0.0	5.9	1.2
	% change-12	−1.2	−22.4	7.1	−3.5	2.4	−8.2
ILMP (73)	Baseline	45 (61.6)	73 (100)	47 (64.4)	23 (31.5)	22 (30.1)	45 (61.6)
	6-month	46 (63.0)	56 (76.7)	43 (58.9)	21 (28.8)	22 (30.1)	36 (49.3)
	12-month	44 (60.3)	45 (61.6)	42 (57.5)	20 (27.4)	21 (28.8)	26 (35.6)
	% change-6	1.4	−23.3	−5.5	−2.7	0.0	−12.3
	% change-12	−1.4	−38.4	−6.8	−4.1	−1.4	−26.0 **
GA + Met (59)	Baseline	47 (79.7)	59 (100)	39 (66.1)	25 (42.4)	22 (37.3)	49 (83.1)
	6-month	46 (78.0)	43 (72.9)	42 (71.2)	24 (40.7)	29 (49.2)	42 (71.2)
	12-month	46 (78.0)	36 (61.0)	37 (62.7)	25 (42.4)	27 (45.8)	38 (64.4)
	% change-6	−1.7	−27.1	5.1	−1.7	11.9 *	−11.9
	% change-12	−1.7	−39.0	−3.4	0.0	8.5 *	−22.4 *

Note: Data presented as *n* (%). % change (6 for 6-month and 12 for 12-month) represents the overall percentage change in respective treatment groups; * and ** represents significant change at *p* < 0.05 and *p* < 0.01 respectively.

**Table 3:** Odds ratio representing the risk of having MetS and its individual components at follow-up compared to baseline in respective groups.

		Baseline	6-Month		12-Month	
		Reference	O.R. (95% C.I.)	*p*	O.R. (95% C.I.)	*p*
**Central Obesity**						
GA	Model a	1.00	0.89 (0.8, 1.0)	0.15	0.94 (0.7, 1.2)	0.65
	Model b	1.00	0.73 (0.5, 1.1)	0.13	0.85 (0.4, 1.7)	0.65
	Model c	1.00	0.71 (0.5, 1.1)	0.12	0.84 (0.4, 1.8)	0.65
ILMP	Model a	1.00	1.06 (0.8, 1.4)	0.65	0.94 (0.7, 1.2)	0.65
	Model b	1.00	1.08 (0.8, 1.5)	0.65	0.92 (0.7, 1.3)	0.654
	Model c	1.00	1.25 (0.5, 3.2)	0.64	0.80 (0.3, 2.2)	0.67
GA + Met	Model a	1.00	0.90 (0.6, 1.3)	0.56	0.90 (0.5, 1.5)	0.71
	Model b	1.00	0.88 (0.6, 1.4)	0.56	0.88 (0.4, 1.7)	0.70
	Model c	1.00	0.74 (0.3, 2.1)	0.56	0.74 (0.2, 3.5)	0.71
**Low HDL-Cholesterol**						
GA	Model a	1.00	1.66 (0.9, 3.2)	0.13	1.53 (0.8, 2.8)	0.18
	Model b	1.00	1.80 (0.8, 3.8)	0.13	1.64 (0.8, 3.4)	0.18
	Model c	1.00	1.83 (0.8, 4.0)	0.13	1.66 (0.8, 3.5)	0.18
ILMP	Model a	1.00	0.75 (0.4, 1.3)	0.34	0.79 (0.4, 1.4)	0.43
	Model b	1.00	0.75 (0.4, 1.4)	0.34	0.79 (0.4, 1.4)	0.43
	Model c	1.00	0.74 (0.4, 1.4)	0.34	0.79 (0.4, 1.4)	0.43
GA + Met	Model a	1.00	1.27 (0.7, 2.4)	0.47	0.86 (0.4, 1.7)	0.67
	Model b	1.00	1.27 (0.6, 2.4)	0.47	0.86 (0.4, 1.7)	0.67
	Model c	1.00	1.27 (0.7, 2.4)	0.47	0.86 (0.4, 1.7)	0.67
**Hypertriglyceridemia**						
GA	Model a	1.00	1.00 (0.7, 1.5)	1.00	0.86 (0.6, 1.3)	0.49
	Model b	1.00	1.00 (0.6, 1.5)	1.00	0.86 (0.6, 1.3)	0.49
	Model c	1.00	1.00 (0.6, 1.6)	1.00	0.86 (0.5, 1.3)	0.49
ILMP	Model a	1.00	0.88 (0.5, 1.5)	0.62	0.82 (0.5, 1.4)	0.44
	Model b	1.00	0.87 (0.5, 1.5)	0.62	0.80 (0.5, 1.4)	0.44
	Model c	1.00	0.86 (0.5, 1.5)	0.62	0.80 (0.5, 1.4)	0.44
GA + Met	Model a	1.00	0.93 (0.6, 1.5)	0.78	1.00 (0.6, 1.7)	1.00
	Model b	1.00	0.93 (0.5, 1.6)	0.78	1.00 (0.6, 1.7)	1.00
	Model c	1.00	0.92 (0.5, 1.6)	0.78	1.00 (0.6, 1.8)	1.00
**Hypertension**						
GA	Model a	1.00	1.46 (0.8, 2.6)	0.19	1.18 (0.6, 2.1)	0.59
	Model b	1.00	1.48 (0.8, 2.7)	0.19	1.18 (0.6, 2.2)	0.59
	Model c	1.00	1.80 (0.8, 4.2)	0.18	1.28 (0.5, 3.1)	0.59
ILMP	Model a	1.00	1.00 (0.7, 1.5)	1.00	0.94 (0.6, 1.4)	0.76
	Model b	1.00	1.00 (0.6, 1.6)	1.00	0.93 (0.6, 1.5)	0.76
	Model c	1.00	1.00 (0.5, 2.2)	1.00	0.88 (0.4, 2.1)	0.76
GA + Met	Model a	1.00	**2.13 (1.1, 4.0)**	**0.019**	**1.86 (1.1, 3.1)**	**0.016**
	Model b	1.00	**2.25 (1.1, 4.5)**	**0.020**	**1.95 (1.1, 3.4)**	**0.017**
	Model c	1.00	**2.85 (1.2, 6.7)**	**0.017**	**2.36 (1.2, 4.6)**	**0.012**
**MetS**						
GA	Model a	1.00	1.06 (0.6, 1.8)	0.83	0.68 (0.4, 1.2)	0.21
	Model b	1.00	1.07 (0.6, 2.0)	0.83	0.64 (0.3, 1.3)	0.21
	Model c	1.00	1.08 (0.6, 2.1)	0.83	0.62 (0.3, 1.3)	0.21
ILMP	Model a	1.00	**0.61 (0.4, 0.9)**	**0.035**	**0.34 (0.2, 0.5)**	**<0.001**
	Model b	1.00	**0.56 (0.3, 0.9)**	**0.033**	**0.29 (0.2, 0.5)**	**<0.001**
	Model c	1.00	**0.52 (0.3, 0.9)**	**0.033**	**0.25 (0.1, 0.4)**	**<0.001**
GA + Met	Model a	1.00	**0.50 (0.3, 0.9)**	**0.049**	**0.37 (0.2, 0.8)**	**0.006**
	Model b	1.00	**0.49 (0.2, 0.9)**	**0.047**	**0.36 (0.2, 0.7)**	**0.006**
	Model c	1.00	**0.46 (0.2, 0.9)**	**0.043**	**0.32 (0.1, 0.7)**	**0.005**

Note: Data presented as O.R. (95% C.I.) for Odds ratio (95% confidence interval). Model “a” is univariate. Model “b” is adjusted at age, sex and BMI at baseline. Model “c” is adjusted with additional covariates like waist, systolic and diastolic BP, fasting glucose, HbA1c, and vitamin D (baseline). Hyperglycemia as a component of MetS is excluded in this analysis as this component is present in all subjects at reference (baseline).

**Table 4:** Intervention effects in number of MetS components and the MetS risk-score.

Treatment Groups				Intervention Effects: Mean Change (95% C.I.), *p*	
	GA	ILMP	GA + Met	ILMP-GA	(GA + Met)-GA
No. of MetS Components					
Baseline	3.15 (0.10)	2.91 (0.11)	3.11 (0.15)	**−0.58 (−0.88, −0.28), <0.001**	−0.12 (−0.53, 0.27), 1.0
6-month	3.14 (0.11)	2.41 (0.12)	2.93 (0.16)		
12-month	2.88 (0.11)	2.10 (0.12)	2.77 (0.16)		
6 M	−0.02 (0.12)	−0.49 (0.13) **	−0.18 (0.17)		
12 M	−0.28 (0.12)	−0.81 (0.13) **	−0.35 (0.18) *		
MetS Risk-score					
Baseline	4.22 (0.08)	4.13 (0.09)	4.28 (0.10)	**−0.31 (−0.53, −0.09), 0.003**	−0.11 (−0.42, 0.19), 1.0
6-month	4.25 (0.07)	3.82 (0.08)	4.22 (0.09)		
12-month	4.12 (0.07)	3.72 (0.08)	4.16 (0.10)		
6 M	0.04 (0.07)	−0.31 (0.07) *	−0.06 (0.09)		
12 M	−0.09 (0.07)	−0.41(0.08) **	−0.12 (0.10)		

Note: Data presented as Mean (Standard error) for baseline, 6 months and 12 months. Changes at time-intervals are presented as mean change (standard error), where 6 M (6-month minus baseline) and 12 M (12-month minus baseline). Overall change in ILMP and GA + Met groups versus GA group are reported as Mean Change (95% confidence interval). Values were adjusted for baseline covariates age, sex and BMI, systolic and diastolic BP, waist, fasting glucose, HbA1c, and vitamin D (baseline values). * represents *p*-value < 0.05 and ** represents *p*-value < 0.01. *p* < 0.05 considered significant.

## Supplementary Materials

The following are available online at http://www.mdpi.com/2072-6643/10/3/383/s1, Figure S1: ROC curve depicting power of MetS_risk factor (RS) for predicting full metabolic syndrome; Table S1: Data showing area under the curve (AUC); Table S2: Data showing coordinates of the ROC curve; Table S3: Odds ratio representing the risk of having metabolic syndrome and its individual components in all samples.
