# Supplementary material for: Effects of Different Dietary and Lifestyle Modification Therapies on Metabolic Syndrome in Prediabetic Arab Patients: A 12-Month Longitudinal Study
Source: Nutrients. 2018 Mar 20;10(3):383. doi: 10.3390/nu10030383 (PMC5872801; doi:10.3390/nu10030383)
Supplement: Supplementary file 1 [file nutrients-10-00383-s001.pdf]

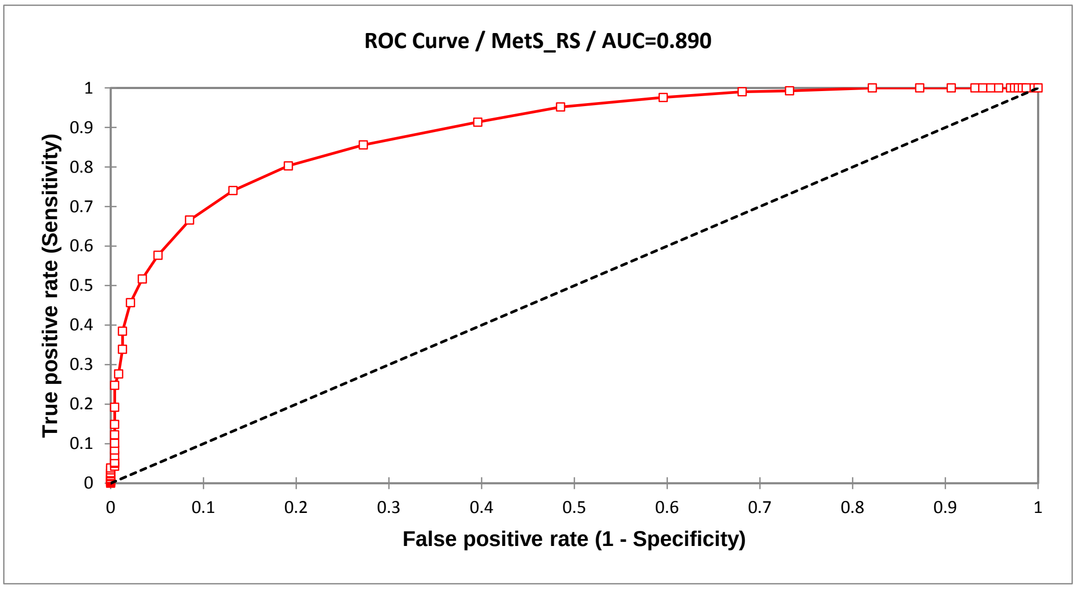

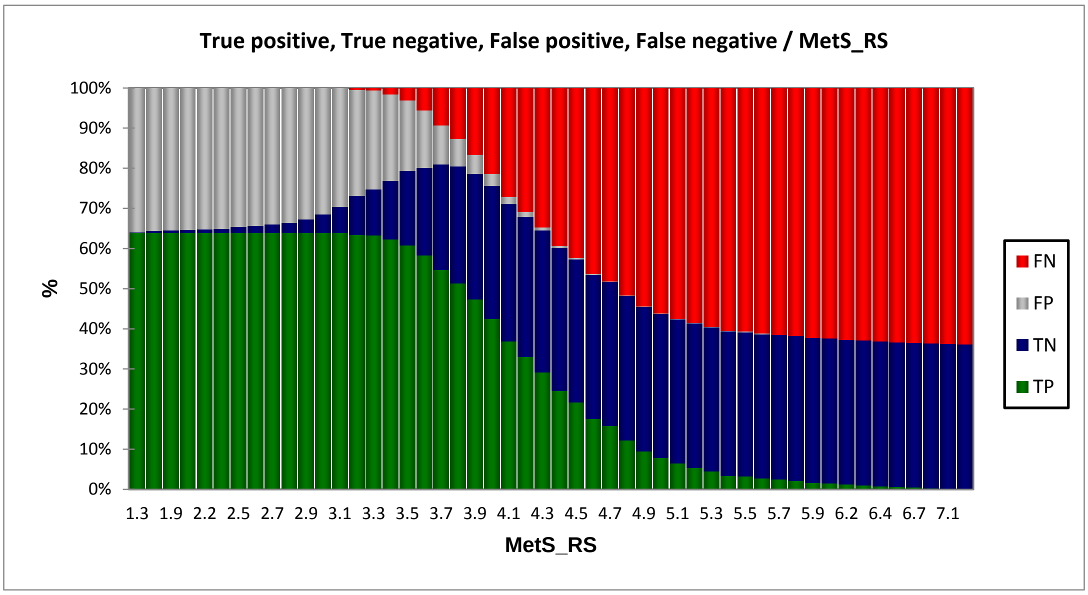

Figure S1. ROC curve depicting power of MetS_risk factor (RS) for predicting full metabolic syndrome.

Table S1: Data showing area under the curve (AUC).

Area under the curve (AUC):				
**AUC**	**Standard error**	**Lower bound (95%)**	**Upper bound (95%)**	**Asymptotic sig [a]**
0.890	0.013	0.865	0.915	.000

[a] Null hypothesis: true area = 0.5

Table S2: Data showing coordinates of the ROC curve

**Coordinates of the Curve**
**Test Result Variable(s):**

Positive if Greater Than or Equal To[a]	Sensitivity	1 - Specificity	Specificity	Youdens max
.3000	1.000	1.000	0.000	0.000
1.4000	1.000	.996	0.004	0.004
1.7000	1.000	.987	0.013	0.013
1.9500	1.000	.983	0.017	0.017
2.1000	1.000	.979	0.021	0.021
2.3000	1.000	.974	0.026	0.026
2.4500	1.000	.970	0.030	0.030
2.5500	1.000	.957	0.043	0.043
2.6500	1.000	.949	0.051	0.051
2.7500	1.000	.940	0.060	0.060
2.8500	1.000	.932	0.068	0.068
2.9500	1.000	.906	0.094	0.094
3.0500	1.000	.872	0.128	0.128
3.1500	1.000	.821	0.179	0.179
3.2500	.993	.732	0.268	0.261
3.3500	.990	.681	0.319	0.310
3.4500	.976	.596	0.404	0.380
3.5500	.952	.485	0.515	0.467
3.6500	.913	.396	0.604	0.518
3.7500	.856	.272	0.728	0.583
**3.8500**	**.803**	**.191**	**0.809**	**0.611**
3.9500	.740	.132	0.868	0.608
4.0500	.666	.085	0.915	0.581
4.1500	.577	.051	0.949	0.526
4.2500	.517	.034	0.966	0.483
4.3500	.457	.021	0.979	0.435
4.4500	.385	.013	0.987	0.372
4.5500	.339	.013	0.987	0.326
4.6500	.276	.009	0.991	0.268
4.7500	.248	.004	0.996	0.243
4.8500	.192	.004	0.996	0.188
4.9500	.149	.004	0.996	0.145
5.0500	.123	.004	0.996	0.118
5.1500	.101	.004	0.996	0.097
5.2500	.084	.004	0.996	0.080
5.3500	.070	.004	0.996	0.065
5.4500	.053	.004	0.996	0.049
5.5500	.050	.004	0.996	0.046
5.6500	.043	.004	0.996	0.039
5.7500	.038	0.000	1.000	0.038

5.8500	.034	0.000	1.000	0.034
6.0000	.026	0.000	1.000	0.026
6.1500	.024	0.000	1.000	0.024
6.2500	.019	0.000	1.000	0.019
6.3500	.017	0.000	1.000	0.017
6.5000	.012	0.000	1.000	0.012
6.6500	.010	0.000	1.000	0.010
6.7500	.007	0.000	1.000	0.007
6.9500	.005	0.000	1.000	0.005
7.6000	.002	0.000	1.000	0.002
9.1000	0.000	0.000	1.000	0.000

The test result variable(s): MetS_RS has at least one tie between the positive actual state group and the negative actual state group. [a]. The smallest cutoff value is the minimum observed test value minus 1, and the largest cutoff value is the maximum observed test value plus 1. All the other cutoff values are the averages of two consecutive ordered observed test values.

Table S3. Odds ratio representing the risk of having metabolic syndrome and its individual components in all samples.

Covariates	Central Obesity	Hyperglycemia	Low HDL-Cholesterol	Hypertriglyceridemia	Hypertension	Full MetS
Age	1.02 (0.9,1.1), 0.382	1.03 (1.01,1.05), 0.012	1.01 (0.9,1.03), 0.636	1.00 (0.97,1.02), 0.880	1.04 (1.01,1.06), 0.005	1.03 (1.00,1.05), **0.043**
Sex	7.01 (3.7,13.1), <0.001	0.42 (0.5,1.3), 0.418	1.68 (1.0,2.7), 0.033	0.5 (0.3,0.8), 0.008	0.76 (0.4,1.3), 0.320	1.68 (1.1,2.7), **0.025**
BMI	1.19 (1.1,1.3), <0.001	1.02 (0.98,1.06), 0.209	1.02 (0.98,1.06), 0.378	1.04 (1.0,1.1), 0.050	1.06 (1.0,1.1), 0.004	1.11 (1.1,1.2), **<0.001**
Waist	1.23 (1.2, 1.3), <0.001	0.99 (0.98, 1.01), 0.934	0.99 (0.97,1.01), 0.534	1.01 (0.99,1.03), 0.209	1.05 (1.02, 1.07), <0.001	1.06 (1.03,1.08), **<0.001**
Systolic BP	1.01 (0.98, 1.03), 0.545	1.00 (0.98, 1.01), 0.980	0.99 (0.97, 1.01), 0.313	1.02 (1.01, 1.03), 0.029	1.14 (1.1, 1.2), <0.001	1.03 (1.01, 1.05), **<0.001**
Disystolic BP	1.02 (0.99, 1.05), 0.171	1.00 (0.98, 1.02), 0.953	0.99 (0.97, 1.02), 0.632	1.02 (0.99, 1.04), 0.095	1.16 (1.1, 1.2), <0.001	1.04 (1.02, 1.06), **0.001**
Fasting Glucose	1.2 (0.7, 2.1), 0.519	1.81 (1.2, 2.8), 0.006	0.69 (0.45, 1.07), 0.102	1.68 (1.06, 2.7), 0.027	2.09 (1.3, 3.4), 0.002	1.97 (1.3, 3.1), **0.003**

Note: Data presented as odds ratio (95% C.I.), p-value. In this table, the risk of age, sex (female versus male) and BMI, waist, Systolic BP, Disystolic BP and fasting glucose (all at baseline) is calculated for predicting metabolic syndrome and its components.
